# Disease phenotypic and geospatial features vary across genetic lineages for Tuberculosis within Arkansas, 2010–2020

**DOI:** 10.1371/journal.pgph.0001580

**Published:** 2023-02-23

**Authors:** Marissa E. Renardy, Craig Gillen, Zhenhua Yang, Leonard Mukasa, Joseph Bates, Russ Butler, Denise E. Kirschner

**Affiliations:** 1 Department of Microbiology and Immunology, University of Michigan Medical School, Ann Arbor, Michigan, United States of America; 2 Department of Biology, AdventHealth University, Orlando, FL, United States of America; 3 Department of Epidemiology, School of Public Health, University of Michigan, Ann Arbor, Michigan, United States of America; 4 Arkansas Department of Health, Little Rock, AR, United States of America; 5 Epidemiology Department in the, Boozman College of Public Health at the University of Arkansas Center for Health Sciences, Little Rock, AR, United States of America; Universidad Autonoma de Baja California, MEXICO

## Abstract

Tuberculosis (TB) elimination in the United States remains elusive, and community-specific, localized intervention strategies may be necessary to meet elimination goals. A better understanding of the genotypic diversity of Mtb, the population subgroups affected by different TB strains, and differences in disease presentation associated with these strains can aid in identifying risk groups and designing tailored interventions. We analyze TB incidence and genotype data from all Arkansas counties over an 11-year time span from 2010 through 2020. We use statistical methods and geographic information systems (GIS) to identify demographic and disease phenotypic characteristics that are associated with different Mtb genetic lineages in the study area. We found the following variables to be significantly associated with genetic lineage (p<0.05): patient county, patient birth country, patient ethnicity, race, IGRA result, disease site, chest X-ray result, whether or not a case was identified as part of a cluster, patient age, occupation risk, and date arrived in the US. Different Mtb lineages affect different subpopulations in Arkansas. Lineage 4 (EuroAmerican) and Lineage 2 (East Asian) are most prevalent, although the spatial distributions differ substantially, and lineage 2 (East Asian) is more frequently associated with case clusters. The Marshallese remain a particularly high-risk group for TB in Arkansas.

## 1. Introduction

Tuberculosis (TB) was the deadliest infectious disease worldwide until the COVID-19 pandemic [1,2] and remains a leader in world-wide deaths. TB is caused by *Mycobacterium tuberculosis* (Mtb), and most commonly affects lungs, though extrapulmonary disease accounts for approximately one quarter of cases in the US [3]. The majority of individuals infected with Mtb develop latent TB infection (LTBI) in which they experience no clinical symptoms. Individuals with LTBI may later develop active TB disease through reactivation of the original infection (endogenous) or through reinfection (exogenous), though the majority of LTBI cases never progress to TB disease [4]. Progression from LTBI accounts for approximately 80% of TB disease cases in the US [5]. Current standards for diagnosis and treatment of TB and LTBI are provided by the Division of Tuberculosis Elimination [6]. In the US, incidence for TB is approximately 2.7 per 100,000 people per year, and it is estimated that 13 million people are living with latent TB infection. While the USA has one of the lowest TB case rates in the world, progress controlling and preventing infection remains too slow to meet TB elimination goals [7,8]. Understanding TB epidemiology in different demographic and geographical settings is critical to designing efficient interventions to reduce TB burden and meet elimination timelines in the US.

The Mtb complex–the group of mycobacterium species that can cause tuberculosis, including Mtb–has evolved several distinct lineages through co-evolution with humans in different geographic regions [9]. There are seven distinct lineages of Mtb Indo-Oceanic, East Asian, East African Indian, Euro-American, two West African lineages, and one occurring primarily in Ethiopia (S1 Table) [10,11]. In addition, humans can be infected with the closely related mycobacterium *M*. *bovis*. Previous studies have found that Mtb lineages have been associated with differences in virulence, immunogenicity, drug resistance, and clinical phenotype [12,13]. For example, Lineage 2 (L2, East Asian) and Lineage 4 (L4, Euro-American) appear to be not only the most wide-spread, but also more virulent than other lineages [10], and lineage 2 has been associated with drug resistance [14,15]. *M*. *bovis* and lineage 3 (L3, East African Indian) have been associated with higher risk of extrapulmonary TB disease [16,17]. Immune responses to Mtb have been shown to vary by lineage, specifically macrophage responses, which likely play a role in the observed differences in rates of disease progression and transmission [18]. Thus, prevalence of various TB genotypes may influence the effectiveness of intervention strategies.

While many studies on TB lineages (cited above) have been performed in high-incidence settings, few have focused on low-incidence settings such as rural states in the USA. In this work, we focus on the state of Arkansas, which has a comprehensive TB data set. AR has lower TB incidence and higher genotype surveillance coverage than the national average. The potential role of microbial factors in TB epidemiology and pathogenesis were reported by several previous association studies conducted in Arkansas to assess the relationship between DNA polymorphism in Mtb genes implicated in TB virulence and the clinical and epidemiological phenotypes of TB [19–21]. A better understanding of the genotypic diversity of Mtb in Arkansas, the population subgroups affected by different TB strains, and differences in disease presentation associated with these strains can aid in identifying risk groups and designing tailored interventions to overcome the slowing decline in TB incidence in recent years.

In this study, we analyze the incidence of different TB lineages in Arkansas during the years 2010–2020. We identify demographic and disease phenotypic factors associated with TB lineages, and perform geospatial analyses using geographic information systems (GIS) to better understand variations in TB prevalence and incidence within and across Arkansas. In particular, we assess socio-demographic and Mtb lineage state-wide patterns and the relationships between these and other factors over both time and geographic space at the scale of ZIP codes. We also compare these results with previous molecular epidemiological studies in a variety of settings. Finally, we discuss the implications of these findings on future TB elimination efforts in Arkansas.

## 2. Methods

### 2.1. Ethics statement

This study used an existing deidentified TB surveillance dataset and involved no additional data collection requiring interactions with the study patients. The study was reviewed and approved by both the University of Michigan Medical School Institutional Review Board (#UMHUM00167846) and the Ethical Committee of the Arkansas Department of Health (#1703679–1) before the start of the research.

### 2.2. Study area

The study area was the USA state of Arkansas (S1 Fig). Arkansas is located in the south-central United States and is bordered by five other states with virtually the entire eastern boundary formed by the Mississippi River (S1 Fig) By area, Arkansas ranks 29th and by population 33rd (out of 50 US states). The population centers are Little Rock, the state’s capital (over 550,000 people in metropolitan area), located in the northwest; Fayetteville, Springdale and Bentonville (headquarters of Walmart, Inc.) with almost 550,000 people and Jonesboro in the northeast; Pine Bluff in the southeast; Texarkana in the southwest; and Fort Smith in the west (see S1 Fig). People identifying as White comprise the largest segment of the population (~70%) followed by Black/African American (~15%), Hispanic (~8.5%), Asian (~1.7%), and native Hawaiian and Pacific Islander (~0.5%) (US Census, 2020).

### 2.3. Study sample and data collection

The study sample included a total of 949 TB cases reported in Arkansas from 2010 to 2020. Of these 949 study cases, 686 (72%) were confirmed by mycobacterial culture and the remaining 263 were based on clinical diagnosis with negative culture. The genetic lineage was identified in 605 cases (64%). Almost all culture positive cases (98.1%) had a genetic lineage identified. Of all sputum culture positive cases, 1.8% did not have a genetic lineage identified. Of all tissue culture positive cases, 2.4% did not have a genetic lineage identified. Of all culture positive cases (sputum and tissue), 1.9% did not have a genetic lineage identified. Both spoligotyping and 24 Loci MIRU-VNTR typing data were available for all the cases included in our study.

Sociodemographic characteristics (e.g. age, sex, race/ethnicity, etc.) and clinical characteristics (previous TB diagnosis, chest X-ray findings, sputum smear and culture results, and HIV infection) were retrieved from the Arkansas TB surveillance data collected by the Arkansas Department of Health (ADH) using the Report of Verified Cases of TB (RVCT) developed by the Centers for Disease Control and Prevention (CDC). Genotyping data of Mtb isolates of the study patients, including SPOLIGOtype, MIRU, state cluster name, and genetic lineage, were drawn from the TB Genotyping Information Management System (TB GIMS). Patient identifiers were removed from the data set before the data were provided to the researchers. The study protocol was approved by the University of Michigan Health Science and Behavior Science Institutional Review Board (HUM00073414), Ann Arbor, MI, USA and the

Arkansas Department of Heath Scientific Advisory Committee.

### 2.4. Study genetic lineage and cluster identification

In this spatial analysis, we define our lineages by geography. Geographic dispersion includes *Mycobacterium tuberculosis* (*M*. *tuberculosis*) (Mtb) lineage (Indo Oceanic) found in areas along the Indian ocean, *M*. *tuberculosis* lineage 2 found majorly in east Asia, *M*. *tuberculosis* lineage 3 found in East Africa and India, *M*. *tuberculosis* lineage 4 (Euro-American) found mainly in Africa, Europe and America, *M*. *tuberculosis* lineage 5 & 6 (*M*. *africanum1* & 2) found exclusively in West Africa and *M*. *tuberculosis* lineage 7 found primarily in Ethiopia [10,11]. (We further clarify that one isolate was typed from each study case, and *study case* here involves analyses of more than one type of case, i.e. clinical cases, culture confirmed, culture confirmed with lineage defined vs those without lineage defined.) There were 81 culture-positive cases not associated with a genetic lineage, either due to unsuccessful genotyping or because the genotype did not match any of the seven major lineages. Individuals were further classified as belonging to a genotypic cluster if their isolates shared an identical genotype based on a combination of identical spoligotype and 24-locus MIRU/VNTR pattern. Genotypically non-clustered TB cases were those not sharing an identical genotype with any other reported TB case in Arkansas as of the end of the study period. This classification was performed at the state level before data were transferred to the researchers.

### 2.5. Statistical analysis of TB case data

All data manipulation and statistical analyses were performed two ways. First, using the program **R** [22] with statistical packages using standard statistical significance that was established using Fisher’s exact test. For age and year arrived in the US, pairwise Wilcoxon test was used. Second, analysis was performed applying geo-statistics using a GIS software package for spatial statistics (Arc Pro 2.8 [23]). Each approach identified a set of significant variables and there was overlap in those identified; however, there were also unique variables to each approach. We discuss this in detail.

#### 2.5a. Standard statistical approaches

TB surveillance and genotyping datasets were merged using the state case number as an identifier. Details on data cleaning can be found in the (S1 Text). We identified categorical variables that significantly varied across different Mtb lineages using Fisher’s exact test (S2 Text). We identified numeric variables that significantly varied across Mtb lineages using a Kruskal-Wallis test, with pairwise significance evaluated using the pairwise Wilcox test (S2 Text). For all statistical tests, we used a significance cutoff of p = 0.05 and we used the Benjamini-Hochberg procedure to correct for multiple testing [24]. In addition to the base R packages, the packages ggplot2 and ggpubr were used to generate figure plots [25,26].

#### 2.5b Geospatial analysis

We utilized GIS to visualize and analyze the spatial distribution of different Mtb lineages in addition to population demographic and socioeconomic features. We used the software package ArcPro 2.8 [23]. Our first step was geocoding, a method of attaching geographic coordinates to features. Features for this analysis include: Arkansas state boundary, cities and its counties, 5-digit Arkansas ZIP codes, and number and lineage of TB cases per ZIP code across the state. Our next step was a process called enrichment, where attribute data are attached as variables to the geocoded layers. Our attribute data included: population and population by race, socioeconomic attributes like house-hold income, federal poverty level (FPL) of 100% or lower, and proportion of no health insurance per ZIP code (Table 1). These attribute data were uploaded and attached directly from the Environmental Systems Research Institute (ESRI) GIS ArcPro 2.8 database. For each map, number of TB cases and normalized demographic data are shown in categories defined by Jenks natural breaks, a data classification method in which categories are based on natural groupings inherent to the data [27,28].

**Table 1:** Independent variables used in exploratory regression. Here, “normalized” refers to variables mapped to values in [0,1] by dividing by the maximum value in the dataset. Proportions are calculated as a proportion of the total population by ZIP code. ESRI was the source of all these data [23].

Independent variable	Data type
2019 Number of people with no health insurance	proportion
2019 Number of households below poverty	normalized
2021 Population density	normalized
2021 Total population	normalized
2021 White population	proportion
2021 Hispanic population	proportion
2021 Black population	proportion
2021 Asian population	proportion
2021 Pacific Islander population	proportion
2021 America Indian population	proportion

Using these demographic and socio-economic metrics, we assessed for possible spatial correlates to the total number and distribution of TB cases observed per ZIP code (2010–2020) using exploratory spatial regression (ESR) and spatial generalized linear regression (GLR). The ESR threshold values were set to a maximum of five explanatory variables, minimum adjusted r^2^ of 0.5, and minimum acceptable spatial autocorrelation p-value of 0.1. Additionally, we used a GLR model (continuous Gaussian/ordinary least squares) with the highest correlating, independent variables found from the ESR to predict the number of TB cases per ZIP code. In addition to these regression techniques, we used an advanced geostatistical procedure, Kriging (S3 Text), to create surface-interpolation maps of predicted total TB cases as a function of associated variables revealed by exploratory regression. This result was displayed as a choropleth image.

The demographic and socio-economic variables ranged over multiple orders of magnitude. In addition, the number of TB cases recorded for the study period (2010–2020) were non-normally distributed. Such variation in datasets can affect statistical fidelity. To address this, we standardized the data in several ways (S1 Text).

Because of personal health information (PHI) our spatial analysis results clustered all ZIP codes recording 0 to six TB cases per ZIP code during the study period. Arkansas contains rural areas with ZIP codes containing small populations; therefore, displaying TB cases with 5 or less reported cases could be of PHI concerns, thus we do not indicate those cases.

## 3. Results

Out of the 949 TB cases reported in Arkansas during 2010–2020, 605 (64%) cases were defined as belonging to one of the seven genetic lineages. Among these cases, 64% (n = 390) were L4 (EuroAmerican), 23% (n = 138) were L2 (East Asian)), 9% were L1 (IndoOceanic) (n = 52), 2% were Bovis (n = 12), and 1% were L3 (East African Indian) (n = 8). In addition, there were 3 cases of Bovis-BCG, which were not included in these analyses since this strain is not human transmissible. Two cases were of mixed lineage, which were also not included in these analyses. We found ten categorical variables to be significantly associated with genetic lineage (p<0.05). These variables are: patient county, occupation risk, patient birth country, patient ethnicity, race, IGRA result, disease site, chest X-ray result, whether the initial treatment regimen included pyrazinamide, and whether or not a case was identified as part of a cluster. Further, we found two numeric variables, patient age and date arrived to the US, to be significant (p<0.05). Below, we organize these features into four categories: spatial and temporal factors, demographic factors, disease phenotype, and transmission.

### 3.1. Overall trends of TB cases in Arkansas- Dominance of Lineage 2

#### 3.1.1. TB temporal trends in Arkansas

**Fig 1:**
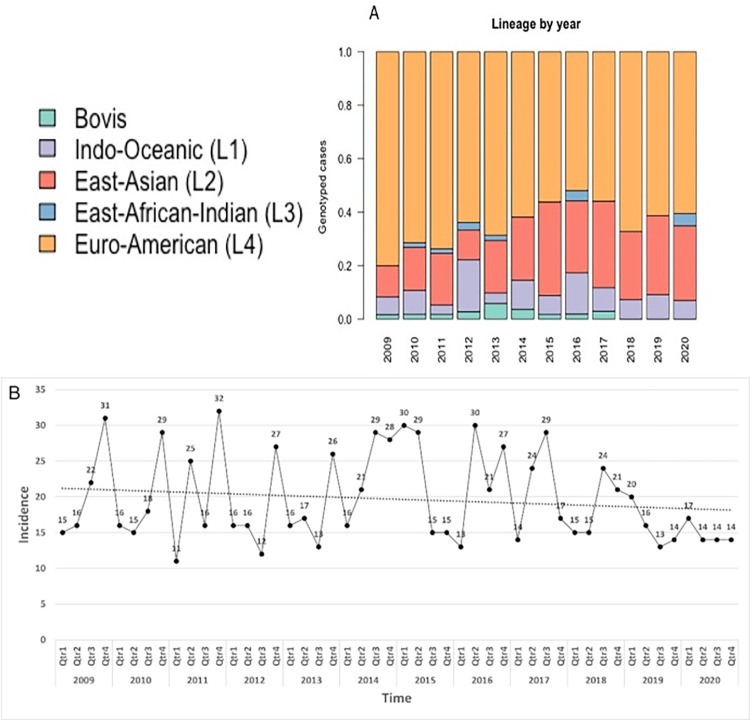
Yearly analysis of TB case load, 2010–2020. (Fig 1A) The proportion of TB lineages per year. (Fig 1B) Incidence of state-wide TB cases per quarter. Note the trend line is not significant (p > .02) indicating the incidence is relatively flat.

The relative proportion of each lineage among incident TB cases remained reasonably consistent over the study period (Fig 1A). Lineage 2, though, increased proportionally early in the study period and then remained constant at approximately 25% of total cases per year (Fig 1A). The temporal trend indicates that the state-wide incidence of all cases during study period remained relatively flat (Fig 1B).

#### 3.1.2. TB spatial patterns in Arkansas- Dominance of Lineage 4 cases

**Fig 2:**
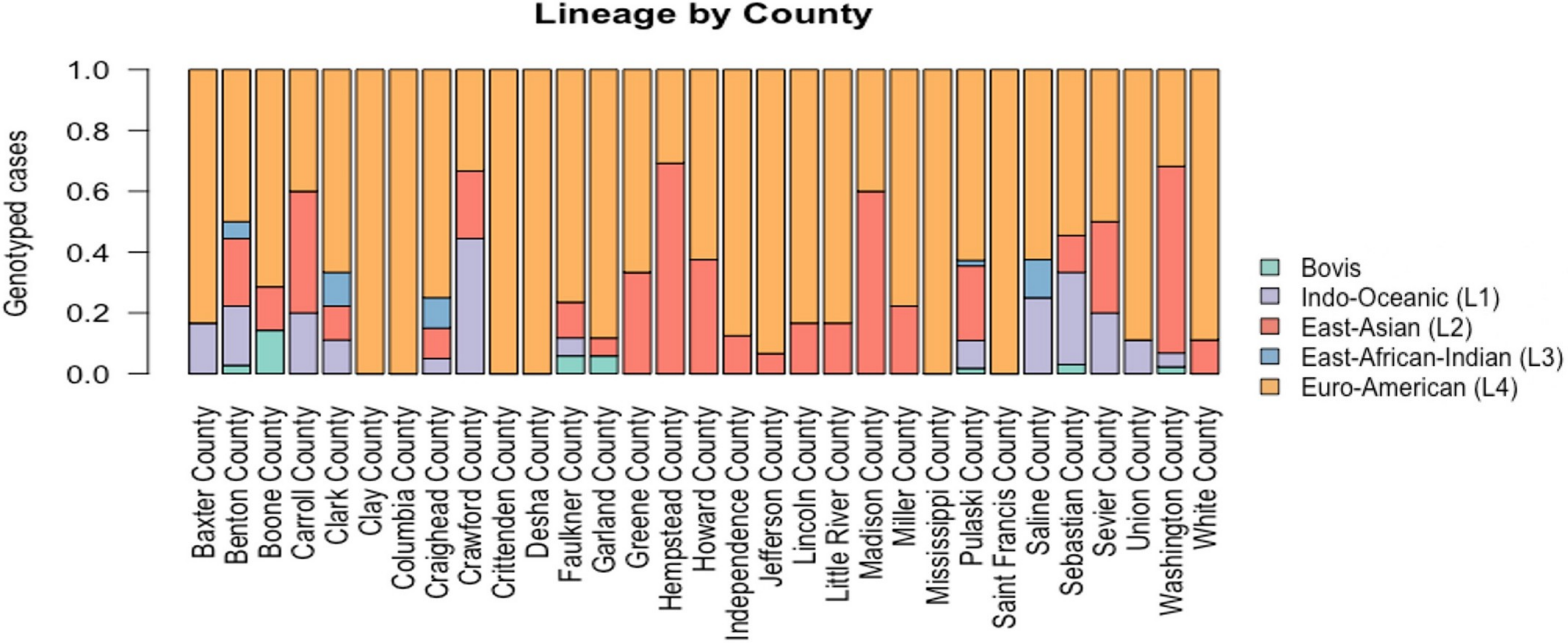
Proportion of genotyped Mtb cases per lineage by Arkansas county for the entire study period. Only those counties (31 in total) with greater than five cases are displayed.

Of the 75 counties within Arkansas, 31 recorded TB cases over the study period with most of the counties reporting multiple lineages present (Fig 2). The most observed was lineage 4. In 21 counties, this lineage was proportionately dominant, with several counties exhibiting only L4 lineage cases (Fig 2).

All ZIP code designations for the geocoding only utilized the 5-digit code. As a result, a total of 579 Arkansas ZIP codes were spatially rendered in the geographic information software. Of this, 211 ZIP codes, or 36.4%, recorded one or more TB cases during the study time frame.

**Fig 3:**
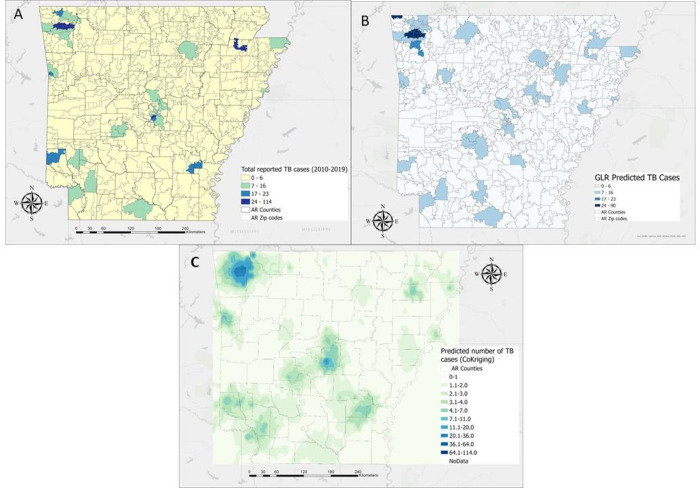
Spatial and geospatial regression analysis of total TB case load in Arkansas during 2010–2020. (Fig 3A) The spatial distribution of total reported TB cases in 2010–2020 in the study area. (Fig 3B) Predicted TB cases by ZIP code using a generalized linear regression model with 4 independent variables, namely total population, proportion of Hispanic and Pacific Islander populations and normalized households below poverty (Table 2). (Fig 3C) Predicted TB cases using Co-Kriging with the same 4 independent variables as in panel B (Table 2). Basemap is Light Gray Canvas. Source: County of Pulaski, AR, Esri, HERE, Garmin, FAO, NOAA, USGS, EPA, NPS. Projection: Mercator Auxiliary Sphere; Datum: D WGS 1984. ZIP Codes: United States ZIP Code Boundaries 2021; Esri, U.S. Census Bureau.

The overall distribution of ZIP codes that recorded TB tended to be spotty across the state (Fig 3). ZIP codes with higher number of TB cases were spread out across the state in three locations where the highest number of cases occurred in the northwest. By region they are distributed by ZIP code as follows: (72764: 114 TB cases and 72762: 24 TB cases), northeast (72401: 26 TB cases) and central (72204: 27 TB cases) portions of the state (Fig 3A).

We performed an exploratory regression on the total population to identify variables that most correlated with the number of TB cases per ZIP code. Our analyses showed a significantly increased risk (+) of TB cases for the proportion of Hispanic (+), the number of households below poverty (+), and the proportion of Pacific Islander population (including Marshallese) (+), with a significantly decreased risk (-) for the proportion of White (-) (Table 2). Additionally, using a regression analysis (GLR) indicates that the inclusion of four variables–total population, proportion of Hispanic and Pacific Islander populations and normalized households below poverty (FPL 100% or less) resulted in an adjR^2^ of 0.77. This means that these 4 variables account for most of the cases identified. Adding more variables only raised the R^2^ value by a few hundredths; although there was some collinearity among the population and socio-economic variables, spatial autocorrelation was not significant (Moran’s I, p > 0.05).

**Table 2:** Results of exploratory regression analysis to identify key variables. Out of all possible regression models including combinations of the 10 explanatory variables, we show the percentage of models for which each variable is significant, negatively correlated, and positively correlated with the number of TB cases per ZIP code.

Variable	% Significant	% Negative	% Positive
2021 Total Population*	100	0	100
2021 Proportion Hispanic Population*	100	0	100
2019 Normalized Households Below Poverty*	95.70	0	100
2021 Proportion Pacific Islander Population*	93.75	0	100
2021 Log10 Population Density	70.70	0	100
2021 Proportion Black Population	67.97	25.78	74.22
2021 Proportion Asian Population	61.72	0	100
2019 Proportion No Health Insurance	45.31	14.06	85.94
2021 Proportion American Indian Population	32.03	42.19	57.81
2021 Proportion White Population	99.61	100	0

The top 4 (indicated by *) are the chosen key variables (adjR^2^ of 0.77).

We then included the four variables we identified with highest correlation described above (Table 2) in the GLR. We used as our cutoff values those variables that totaled over 90% significant and were positively correlated (Table 2). The GLR results indicate that these variables were relatively strong predictors of TB cases per ZIP code (Fig 3B). The GLR predicted ZIP Codes with 0–6 cases to be distributed similarly to the case-data distributions (Fig 3A & 3B). Additionally, the GLR model was a good predictor of areas with relatively high TB prevalence (Fig 3A & 3B). However, as might be expected with complex dynamics of infectious diseases, the GLR model under-estimated the highest count of TB-cases (90 vs 114) (Fig 3A & 3B).

One strong analytic advantage to having georeferenced data is that from geographic locations of the phenomena of interest, an entire surface can be interpolated. The results of an additional geo-spatial analysis called Co-Kriging Surface Analysis (Fig 3C) indicates that utilizing the same four variables included in the GLR, constructed a TB, spatial-distribution pattern similar to the results of the ZIP-code based spatial regression model. However, a feature of Co-Kriging analysis is that the resulting output is independent of artificially created boundaries (e.g., ZIP codes/counties), which constrains the regression model, but creates isopleth boundaries of the magnitude and spatial pattern of the location data (Fig 3B & 3C). A key feature of these geostatistical derived boundaries that are not corresponding to standardized population or government spatial units, is that data can be displayed in these images that would have been identifiable if displayed using census or postal boundary layers but is not in this approach (Fig 3B & 3C).

#### 3.1.3. Distributions of lineages

**Fig 4:**
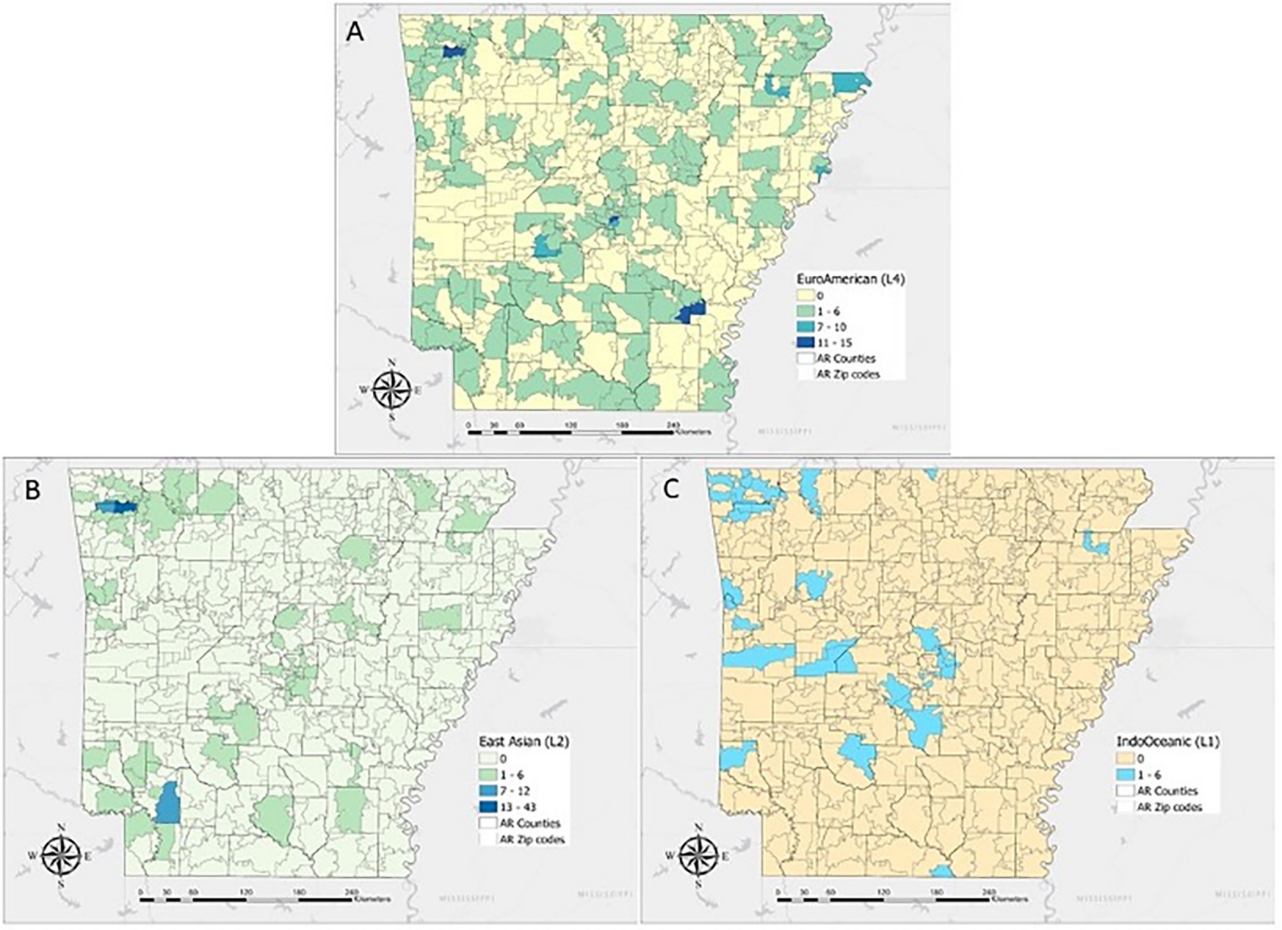
Spatial analysis by TB lineage for Arkansas. (Fig 4A) Distribution of Lineage 4 (L4) (Fig 4B) Distribution of Lineage 2 (L2). (Fig 4C) Distribution of Lineage 1 (L1). The frequencies, per ZIP code, for other lineages were too low to be displayed per HIPAA. We omit the East African Indian and Bovis lineages due to their small sample size. Basemap is Light Gray Canvas. Source: County of Pulaski, AR, Esri, HERE, Garmin, FAO, NOAA, USGS, EPA, NPS. Projection: Mercator Auxiliary Sphere; Datum: D WGS 1984. ZIP Codes: United States ZIP Code Boundaries 2021; Esri, U.S. Census Bureau.

The prevalence of different lineages among TB cases varied significantly across ZIP codes and counties of residence. We show the distribution of Mtb lineage by ZIP code over the study period in Fig 4, for L4, L2, and L1. Of note is that lineage L4 was present with particularly high prevalence in areas of Garland and Jefferson Counties (85% and 92% of genotyped cases, respectively) and a particularly low prevalence in Washington County (29% of genotyped cases). Additionally, lineage L2 was present with particularly high prevalence in the Springdale/Fayetteville area in Washington County (63% of genotyped cases). Lineage 1 is mostly concentrated in the Fort Smith area in Sebastian County, where it accounted for 36% of genotyped cases.

#### 3.1.4. The Relationship between Demographics and TB lineages

We explore key demographic population features and their relation to TB lineage cases. For this analysis, we focus on age, race, and immigrant status. Patient ethnicity indicates hispanic or non-hispanic, whereas race indicates white/black/asian/etc.

*Age*: *M*. *bovis* infections occurred in patients significantly older than patients with other lineages. Patients infected with *M*. *bovis* were 65.8 years old on average, while the average age of all TB patients was 47.3 years. Patients infected with lineage L3 (mean 33 years) were significantly younger than those infected with Lineage 1 (mean 46.1 years) or L4 (mean 54.7 years). Patients whose TB lineage is unknown (culture negative and clinical cases included) (mean 40.4 years) were also significantly younger than those who were associated with a TB lineage (mean 51.4 years). We show the age distributions for each of the TB lineages, as well as for cases not associated with a lineage (S2 Fig).

*Race/Ethnicity*: Patients with genotype data available who were Asian had the highest prevalence of lineage 1 (56% vs. 9% overall prevalence). Patients who were American Indian or Alaska Native, Black/African American, or White had a high prevalence of lL4 (100%, 76%, and 82%, respectively, vs. 65% overall prevalence). Of the Black or African American patients who were genotyped, a relatively high proportion (23%) were infected with L2, and these patients were primarily US-born. Patients who were Native Hawaiian or Other Pacific Islander had the highest prevalence of the L2 (86% vs. 23% overall prevalence). Lineage 4 was more prevalent in Hispanic or Latino TB patients (79% vs. 63% for non-Hispanic), while L2 was more prevalent in non-Hispanic/Latino TB patients (26% vs. 3% for Hispanic/Latino). We summarize the breakdown of TB cases for each race by lineage in S2 Fig.

GIS-based population distribution maps indicate that both Asian and Pacific Islander populations do not exceed 15% of the population for any ZIP code, and both tend to cluster in the northwestern corner of the state (S3 Fig). White and Black populations appear more evenly distributed across the study areas and are inversely related (S3A and S3B Fig). The Asian and Pacific Islander population distribution spatially coincides with L2 TB lineage (Fig 4B). We note the relatively high proportion of Pacific Islanders in the Springdale/Fayetteville area is largely comprised of immigrants from the Marshall Islands (S3D Fig).

*Immigrant status*: To investigate the effects of immigration, we separated the population of TB patients into those born in the US vs. those born outside the US. Among all genotyped TB cases in our dataset, 68% occurred in US-born individuals. We found that infections with Bovis and TB lineage 4 primarily occurred in US-born individuals (83% and 82%, respectively), while infections with L1 and L3 lineages primarily occurred in non-US-born individuals (100% and 79%, respectively). Infections with L2 were almost evenly split between US-born and non-US-born individuals (51% non-US-born, 49% US-born). We found that time since arriving in the US was generally not significantly different across lineages. The only significant difference in how long non-US-born TB patients had been in the US was between lineages L1 and L2 (p<0.05). Those infected with lineage 1 arrived in the US on average 5.6 years earlier than those infected with lineage 2. We summarize the breakdown of immigrant status and date arrived in the US by TB lineage in S4 Fig, and highlight the distribution of overall proportions foreign-born individuals (S4 Fig).

**Fig 5:**
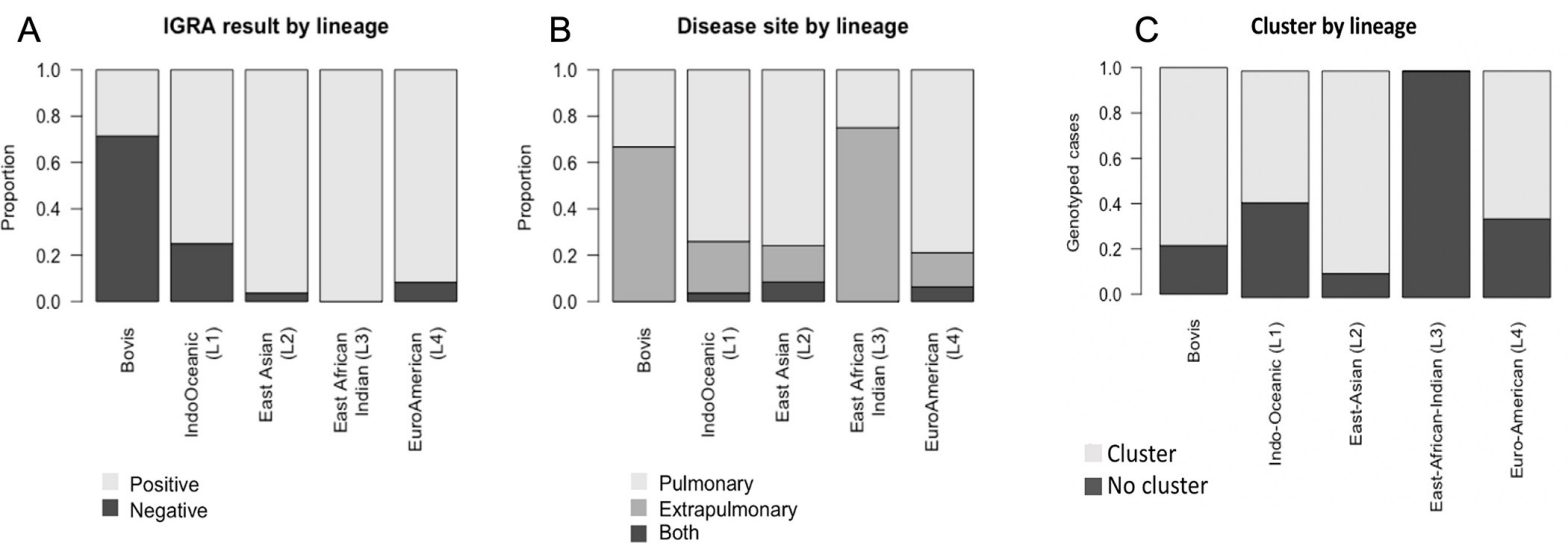
Disease phenotypic factors by TB lineage. (Fig 5A) Proportions of positive and negative IGRA results by lineage, among genotyped TB cases with a determinate IGRA result (positive/negative). (Fig 5B) Proportions of TB cases that were diagnosed as pulmonary, extrapulmonary, or both by lineage. (Fig 5C) shows the distribution of TB cases associated with clusters by lineage. Shown for each lineage is the proportion of genotyped TB cases that are associated with a state genotypic cluster.

*3*.*1*.*4*.*1 Disease phenotype*. *IGRA result*: We found that infections from Bovis lineages were observed to be IGRA negative (60% negative). Individuals infected with lineages L1 or L4 were also observed to be IGRA negative (23% and 18%, respectively) than lineages L2 and L3 (9% and 0%, respectively). IGRA negative cases were uncommon overall, accounting for 17% of genotyped cases with a determinate IGRA result (Fig 5A).

*Disease site(s)*: 81% of all genotyped cases were exclusively pulmonary, while 13% were exclusively extrapulmonary. TB cases that were both pulmonary and extrapulmonary were rare, accounting for only 6% of genotyped cases. Bovis lineage infections were more likely to be extrapulmonary (58%), while other lineages were more likely to be pulmonary (79% for L1, 83% for L2, 58% for L3, and 82% for L4) (Fig 5B).

*Chest X-ray results by lineage*: X-ray detection exhibited 63% abnormal results for the Bovis lineage, 75% for lineage 3, 78% for lineage 1, and over 90% for lineages L2 and L4. (S5 Fig).

#### 3.1.5. Genotypic clusters

The AR dataset included cases associated with 114 clusters containing more than one isolate. For all genotyped TB cases in the dataset, 69% were associated with genotypic clusters. We found that a particularly large fraction of patients infected with lineage 2 were associated with clusters (89%) (Fig 5C). There were 13 clusters associated with lineage 2 cases. None of the four cases of lineage 3 were associated with a cluster. We identified 65% of L4 cases and 58% of lineage 1 cases associated with clusters, with 91 clusters associated with L4 cases and 11 clusters associated with lineage 1 cases. 11 out of 14 *M*. *bovis* cases were associated with only two clusters.

## 4. Discussion

We have analyzed a rich dataset from the Arkansas Department of Public Health consisting of reporting and genotyping data that spans an 11-year time frame and hundreds of patients. We used a novel approach pairing traditional statistical analyses together with geo-spatial analyses yielding results that highlight both temporal and spatial patterns of disease. Here, we focused on differences between TB lineages, their prevalence in different subpopulations, and their impact on disease phenotype. We found that Mtb genetic lineage is differentially distributed by patients’ county, age, race, ethnicity, immigrant status, IGRA result, and disease site. TB lineage was also associated with whether or not cases were identified as part of a genotypic cluster in Arkansas.

The high proportion of the EuroAmerican (L4) and East Asian (L2) lineages among TB cases indicate that these lineages are likely endemic and widespread within Arkansas. A majority of TB cases associated with L4 (EuroAmerican), L2 (East Asian), L1 (IndoOceanic), and Bovis lineages were linked to clusters. By contrast, the few TB cases associated with the L3 (East African) lineage were not linked to any cluster and occurred only in non-US-born individuals; we thus believe these cases likely are derived from reactivation of latent disease originally acquired abroad. TB cases not associated with a genetic lineage were significantly younger patients than those individuals that were associated with a lineage. This suggests that younger TB patients were more likely to be diagnosed based on clinical symptoms. This is not surprising as children with TB most often do not raise sputum so material for culture is not available. The TB diagnosis is typically made by findings on Chest X-ray, symptoms and a history of close contact with an infectious case.

For a population like Arkansas, the wide-spread identification of the L4 (Euro-American) lineage should be expected. We found that a particularly large proportion of L2 (East Asian) lineage TB cases were identified as part of a genotypic cluster (multiple TB cases with identical genotype). This is consistent with previous findings that the L2 (East Asian) lineage has been associated with increased transmission [29–33]. Increased transmission of the L2 lineage is further supported by the relatively high prevalence of the L2 lineage in Black and African American patients who are primarily US-born, as these patients are unlikely to have acquired Mtb infection abroad; African American patients have a known association with greater risk of transmission [34]. It is important to note, however, that genotypic clusters do not necessarily imply recent transmission, particularly in rural settings such as found in regions of Arkansas [35]. While these cases are genetically closely related, we do not include information about the timing of infection, and thus active transmission cannot necessarily be inferred [36,37]. Of importance is that the L2 lineage includes the famous "Beijing family strain’ [38]. This may explain some of the prevalence of disease with this lineage. Finally, some of the larger clustered cases may be associated with employment in the poultry processing industry in those counties, suggesting that screenings in this industry may help identify and isolate spread.

For example, as evidenced by the spatial distribution and high prevalence among Pacific Islanders of the L2 lineage in our dataset, this lineage disproportionately affects the Marshallese population, a population that suffers from high incidence of many chronic diseases within Arkansas [39]. Overall, the proportion of Pacific Islanders, Hispanic Population and the number of individuals living below poverty were the largest predictors of cumulative TB incidence in these counties over the study period. This highlights the importance of focused efforts on these at-risk populations. Particularly, it has been estimated that 50% of the Marshallese population in Arkansas are uninsured [39], a rate much higher than has been reported for the overall Arkansas population, closer to 10% [40]. Given the strong link between the Marshallese population and TB cases in Arkansas, particularly the L2 lineage, interventions increasing access to medical care for this demographic are likely crucial to reverse the current trends. This further supports studies that suggest control measures should account for lineages [41].

We note that genotype-based analysis can generate additional information on the lineages herein. The main purpose of this present study was not to provide an as detailed molecular and clinical characterization of our isolate collection as current technology would allow. Instead, it was to extend the knowledgebase of clinical and epidemiological characteristics of the well-defined major genetic lineages of Mth by analyzing existing population-based TB surveillance and related MTB isolate genotyping data. The major genetic lineages of Mtb have been widely used to describe the global Mtb population structure and molecular epidemiology of TB. *In vitro* and *in animal* studies have suggested lineage-related virulence and found that the deletion of certain genomic region of MTB as the basis for the success in wide spread of some modern lineages of Mtb, including L2, L3, and L4. However, studies that provide a clinical and epidemiological characterization of Mtb genetic lineages using population-based patient data, like our current study, are limited. We believe that a better understanding of the clinical and epidemiological phenotypes of these major genetic lineages can inform of future development of vaccine, diagnostics, and treatment. In addition, we did not attempt the genotype-based analysis as sample sizes of each genotype are too small to have sufficient statistical power to detect potential existing associations.

Importantly, this dataset which accounts for the total TB case load in Arkansas over the eleven-year period 2010–2020, indicates a decrease in TB incidence that is not statistically significant. Statewide case rates have stagnated over this time period [42]. Different origins of disease and disease phenotypes may require additional strategies to optimize resources, and such information could be partially inferred from lineage. Increased understanding of the different types of TB in different communities could allow for more targeted intervention strategies that are necessary to meet TB elimination target timelines.

## Supporting information

S1 Fig Administrative subdivisions of the state of Arkansas relevant to our analyses are counties and ZIP codes. Counties are larger geographic and population regions than ZIP codes and generally are the primary legal and functioning governmental units of a US state. There are 75 counties in Arkansas that average 1836 Sqkm. ZIP code is an acronym for: Zone Improvement Plan and is a type of U.S. postal code to aid the United States Postal Service (USPS) to route mail more precisely and efficiently. ZIP codes were introduced in 1963 and consist of a five-digit designation. The goal of ZIP codes was to divide areas of the country into units smaller than counties and to be reasonably similar to each other with respect to both population size and spatial extent across the nation. ZIP codes across Arkansas averaged 246.5 Sqkm and 5696 people. Basemap is Light Gray Canvas. Source: County of Pulaski, AR, Esri, HERE, Garmin, FAO, NOAA, USGS, EPA, NPS. Projection: Mercator Auxiliary Sphere; Datum: D WGS 1984. ZIP Codes: United States ZIP Code Boundaries 2021; Esri, U.S. Census Bureau.
(TIFF)

S2 Fig Demographic breakdown of TB cases by lineage compared with total population. (S2A) Boxplots demonstrating variability of patient age across different TB lineages. (S2B) Prevalence of different TB lineages among genotyped TB cases by patient race. (S2C) Distribution of racial groups among ZIP Codes that reported TB cases during the study period.
(TIFF)

S3 Fig Distribution of racial/ethnic groups reporting TB. (S3A) Proportion of Black residents. (S3B) Proportion of White residents. (S3C) Proportion of Asian residents. (S3D) Proportion of Pacific Islander residents. (S3E) Proportion of Hispanic residents. Basemap is Light Gray Canvas. Source: County of Pulaski, AR, Esri, HERE, Garmin, FAO, NOAA, USGS, EPA, NPS. Projection: Mercator Auxiliary Sphere; Datum: D WGS 1984. ZIP Codes: United States ZIP Code Boundaries 2021; Esri, U.S. Census Bureau.
(TIFF)

S4 Fig Breakdown of TB lineages by immigration factors compared with total population. (S4A) Boxplots showing variation in date arrived in the US among immigrants diagnosed with TB by lineage. (S4B) Spatial (GIS) images showing proportions of US-born and foreign-born individuals in the study area by county (ZIP Code scale not available). Basemap is Light Gray Canvas. Source: County of Pulaski, AR, Esri, HERE, Garmin, FAO, NOAA, USGS, EPA, NPS. Projection: Mercator Auxiliary Sphere; Datum: D WGS 1984. ZIP Codes: United States ZIP Code Boundaries 2021; Esri, U.S. Census Bureau.
(TIFF)

S5 Fig Chest X-Ray by Lineage. Upper Panel shows the number of abnormal chest X-rays by lineage. A high proportion of patients had abnormal X-rays.
(TIFF)

S1 Table Summary of TB lineages and their characteristics. Lineages are listed in order of prevalence in our dataset. We include the West African (L5-L6) and Aethiops vetus, lineage 7 for completeness, but these lineages are not present in our dataset. Total cases were 949, Number with lineage: 605 (63.8%), Number with no lineage: 344 (36.2%). Cases with Clinical diagnosis: 263, Positive culture but no lineage: 82, Spoligotype but no lineage: 32. Note three were 15 total Bovis-related cases, 3 of which were due to Bovis-BCG. Finally, 2 cases were categorized as Other/mixed.
(DOCX)

S1 Text Data cleaning. (DOCX)

S2 Text Categorical factors associated with TB lineage in Arkansas. (DOCX)

S3 Text Details of geospatial analysis. (DOCX)
