# Supplementary figures and images for: Disease phenotypic and geospatial features vary across genetic lineages for Tuberculosis within Arkansas, 2010–2020
Source: PLOS Glob Public Health. 2023 Feb 23;3(2):e0001580. doi: 10.1371/journal.pgph.0001580 (PMC10022325; doi:10.1371/journal.pgph.0001580)

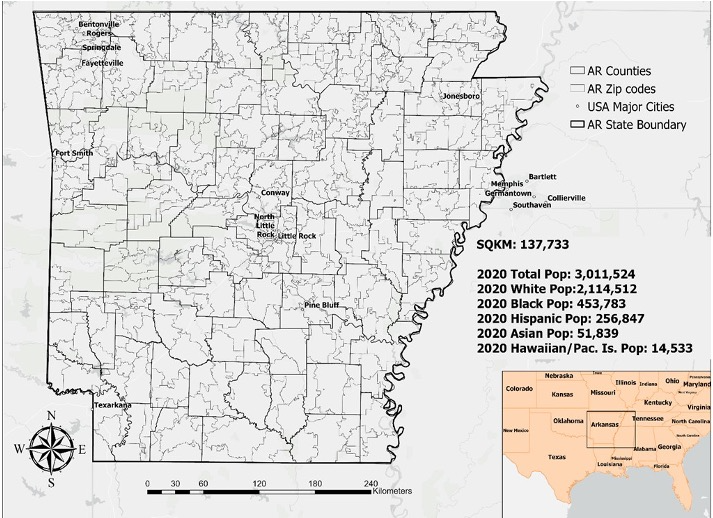

Supplement: S1 Fig — Counties are larger geographic and population regions than ZIP codes and generally are the primary legal and functioning governmental units of a US state. There are 75 counties in Arkansas that average 1836 Sqkm. ZIP code is an acronym for: Zone Improvement Plan and is a type of U.S. postal code to aid the United States Postal Service (USPS) to route mail more precisely and efficiently. ZIP codes were introduced in 1963 and consist of a five-digit designation. The goal of ZIP codes was to divide areas of the country into units smaller than counties and to be reasonably similar to each other with respect to both population size and spatial extent across the nation. ZIP codes across Arkansas averaged 246.5 Sqkm and 5696 people. Basemap is Light Gray Canvas. Source: County of Pulaski, AR, Esri, HERE, Garmin, FAO, NOAA, USGS, EPA, NPS. Projection: Mercator Auxiliary Sphere; Datum: D WGS 1984. ZIP Codes: United States ZIP Code Boundaries 2021; Esri, U.S. Census Bureau. (TIFF) [file pgph.0001580.s001.tiff]

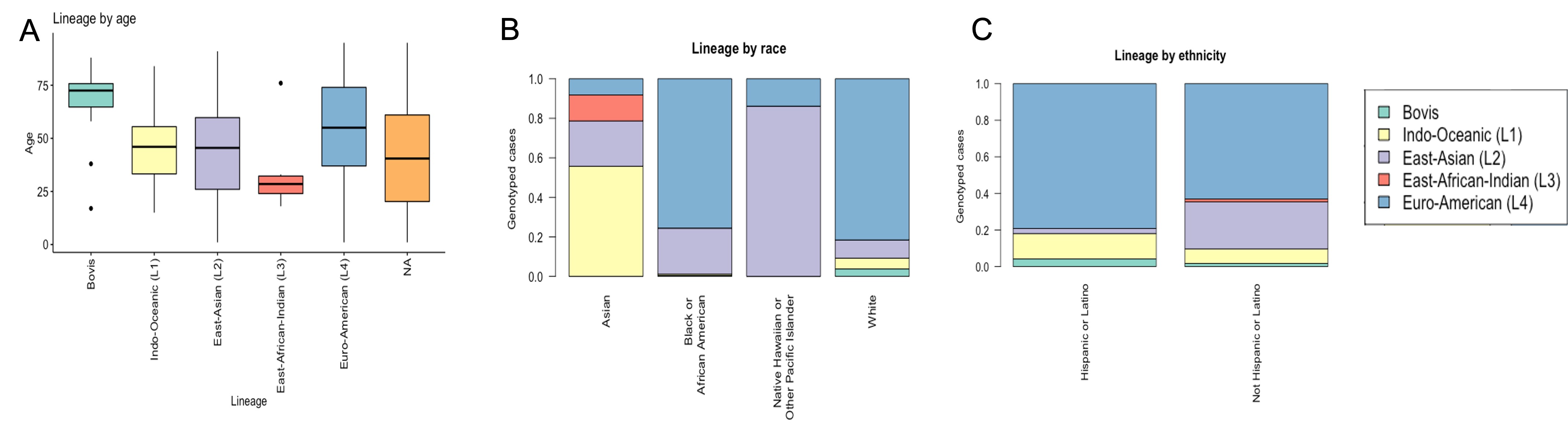

Supplement: S2 Fig — (S2A) Boxplots demonstrating variability of patient age across different TB lineages. (S2B) Prevalence of different TB lineages among genotyped TB cases by patient race. (S2C) Distribution of racial groups among ZIP Codes that reported TB cases during the study period. (TIFF) [file pgph.0001580.s002.tiff]

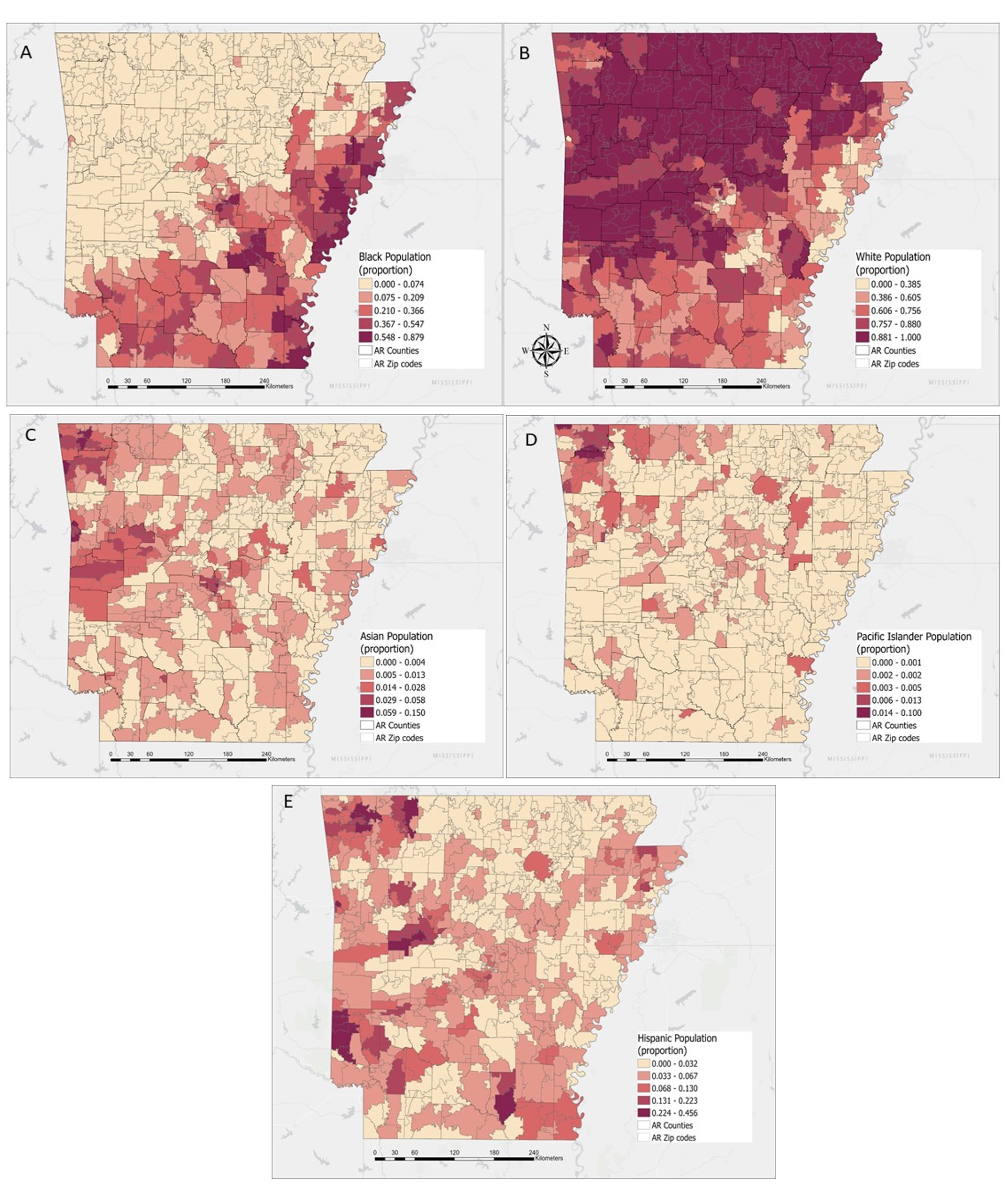

Supplement: S3 Fig — (S3A) Proportion of Black residents. (S3B) Proportion of White residents. (S3C) Proportion of Asian residents. (S3D) Proportion of Pacific Islander residents. (S3E) Proportion of Hispanic residents. Basemap is Light Gray Canvas. Source: County of Pulaski, AR, Esri, HERE, Garmin, FAO, NOAA, USGS, EPA, NPS. Projection: Mercator Auxiliary Sphere; Datum: D WGS 1984. ZIP Codes: United States ZIP Code Boundaries 2021; Esri, U.S. Census Bureau. (TIFF) [file pgph.0001580.s003.tiff]

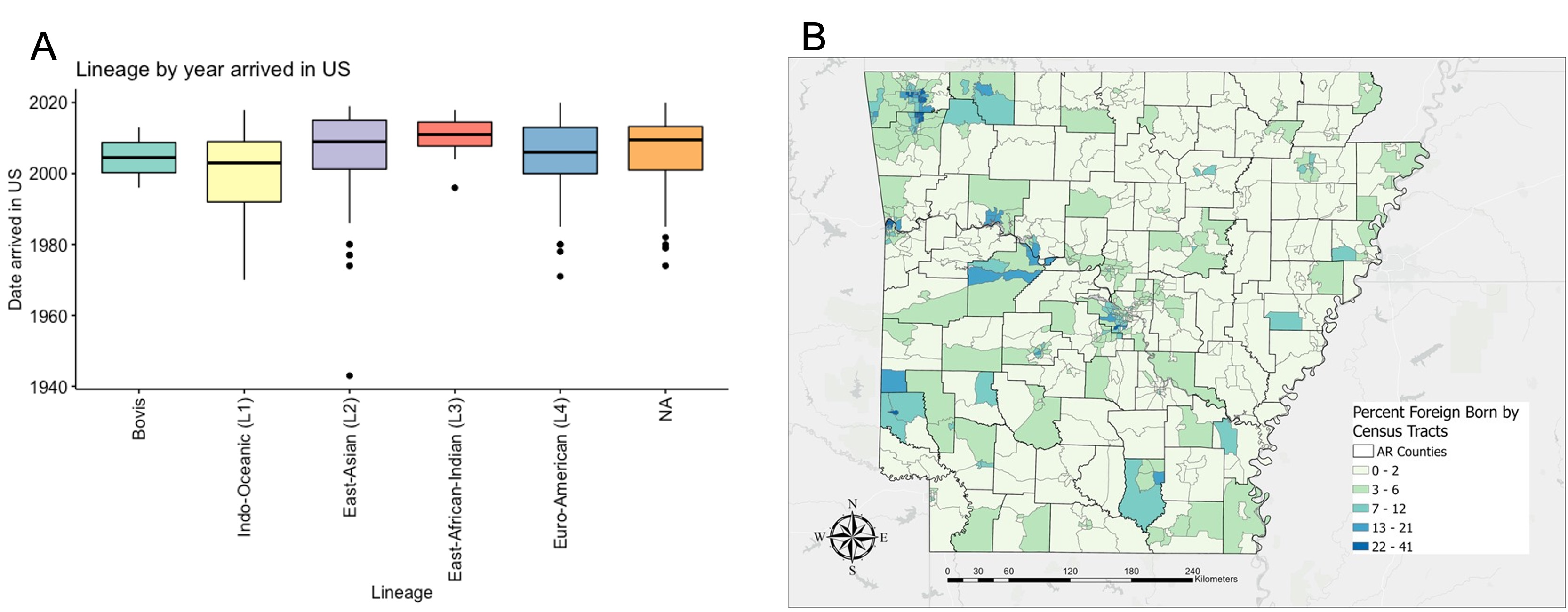

Supplement: S4 Fig — (S4A) Boxplots showing variation in date arrived in the US among immigrants diagnosed with TB by lineage. (S4B) Spatial (GIS) images showing proportions of US-born and foreign-born individuals in the study area by county (ZIP Code scale not available). Basemap is Light Gray Canvas. Source: County of Pulaski, AR, Esri, HERE, Garmin, FAO, NOAA, USGS, EPA, NPS. Projection: Mercator Auxiliary Sphere; Datum: D WGS 1984. ZIP Codes: United States ZIP Code Boundaries 2021; Esri, U.S. Census Bureau. (TIFF) [file pgph.0001580.s004.tiff]

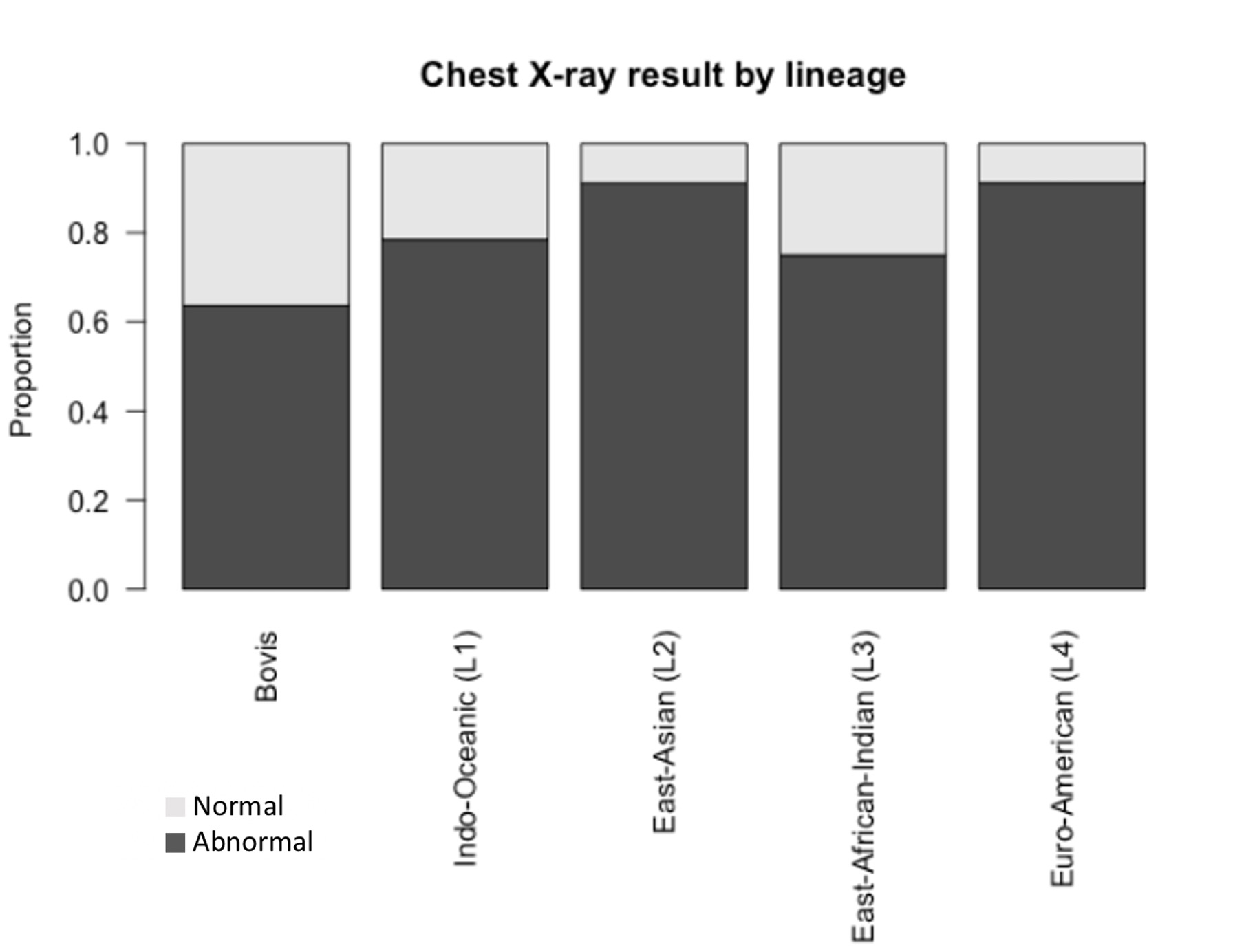

Supplement: S5 Fig — Upper Panel shows the number of abnormal chest X-rays by lineage. A high proportion of patients had abnormal X-rays. (TIFF) [file pgph.0001580.s005.tiff]
