# Supplementary material for: Disease phenotypic and geospatial features vary across genetic lineages for Tuberculosis within Arkansas, 2010–2020
Source: PLOS Glob Public Health. 2023 Feb 23;3(2):e0001580. doi: 10.1371/journal.pgph.0001580 (PMC10022325; doi:10.1371/journal.pgph.0001580)
Supplement: S1 Table — Lineages are listed in order of prevalence in our dataset. We include the West African (L5-L6) and Aethiops vetus, lineage 7 for completeness, but these lineages are not present in our dataset. Total cases were 949, Number with lineage: 605 (63.8%), Number with no lineage: 344 (36.2%). Cases with Clinical diagnosis: 263, Positive culture but no lineage: 82, Spoligotype but no lineage: 32. Note three were 15 total Bovis-related cases, 3 of which were due to Bovis-BCG. Finally, 2 cases were categorized as Other/mixed. (DOCX) [file pgph.0001580.s006.docx]

| **Lineage** | **Counts by Lineage**  **in study** |
| --- | --- |
| L4 (EuroAmerican) | 390 |
| L2 (East Asian) | 138 |
| L1 (IndoOceanic) | 52 |
| L3 (East African Indian) | 8 |
| L5-6 (West African) | 0 |
| L7 Aethiops vetus) | 0 |

**S1 Table: Summary of TB lineages and their characteristics.** Lineages are listed in order of prevalence in our dataset. We include the West African (L5-L6) and Aethiops vetus, lineage 7 for completeness, but these lineages are not present in our dataset. Total cases were 949, Number with lineage: 605 (63.8%), Number with no lineage: 344 (36.2%). Cases with Clinical diagnosis: 263, Positive culture but no lineage: 82, Spoligotype but no lineage: 32. Note three were 15 total Bovis-related cases, 3 of which were due to Bovis-BCG. Finally, 2 cases were categorized as Other/mixed.
