# Supplementary material for: Disease phenotypic and geospatial features vary across genetic lineages for Tuberculosis within Arkansas, 2010–2020
Source: PLOS Glob Public Health. 2023 Feb 23;3(2):e0001580. doi: 10.1371/journal.pgph.0001580 (PMC10022325; doi:10.1371/journal.pgph.0001580)
Supplement: S1 Text — (DOCX) [file pgph.0001580.s007.docx]

**S1 Text. Data cleaning**

Inconsistencies in the study data, such as misspellings or alternative formats, were identified and corrected manually in R. Duplicate data (i.e., multiple genotyping data for the same case number and with the same lineage, such as in cases with multiple disease sites where multiple samples were obtained) were removed using the distinct() function from the dplyr R package (Wickham et al., 2020). Five ZIP Code identifiers in the dataset were either not valid identifiers or entry errors such that the ZIP Codes did not match the target AR TB counties. These data points were removed from the geospatial analyse
