# Supplementary material for: Disease phenotypic and geospatial features vary across genetic lineages for Tuberculosis within Arkansas, 2010–2020
Source: PLOS Glob Public Health. 2023 Feb 23;3(2):e0001580. doi: 10.1371/journal.pgph.0001580 (PMC10022325; doi:10.1371/journal.pgph.0001580)
Supplement: S2 Text — (DOCX) [file pgph.0001580.s008.docx]

**S2 Text.** **Categorical Factors associated with TB lineage in Arkansas**

For the categorical variables, significance was established using Fisher's exact test). For age and year arrived in the US, we used a pairwise Wilcoxon test (similar to a t-test). These are both statistical tests to compare the populations and do not involve regression analysis. Each factor was considered independently.

Significant variables identified using **R**:

- Demographics:
  - Patient county
  - Occupation risk
  - Birth country / immigrant status
  - Ethnicity
  - Race
  - Age
  - Year arrived in US
- Disease phenotype:
  - Disease site
  - Chest x-ray result
  - IGRA result
  - Initial regimen: pyrazinamide
  - Cluster
