# Supplementary material for: Disease phenotypic and geospatial features vary across genetic lineages for Tuberculosis within Arkansas, 2010–2020
Source: PLOS Glob Public Health. 2023 Feb 23;3(2):e0001580. doi: 10.1371/journal.pgph.0001580 (PMC10022325; doi:10.1371/journal.pgph.0001580)
Supplement: S3 Text — (DOCX) [file pgph.0001580.s009.docx]

**S3 Text. Details of geospatial analysis**

GIS is a comprehensive system: digital, technological, analytical and conceptual for gathering, managing, and analyzing data. GIS geostatistical analytics are grounded in geographical sciences and as such, GIS can integrate a wide array of data types. In so doing, GIS technology can layer huge amounts of spatio-temporally linked information into organized visualizations, pertinent associations, and/or important explanatory clusters that can reveal deeper insights into data that can indicate patterns, relationships and situations. Esri©, which created and supports the ArcPro GIS platform, collects and curates terabytes of global environmental, demographic, socio-economic, remotely sensed, and human health information into spatio-temporal datasets that can be integrated into GIS analytics.

ZIP codes can have some peculiarities. For example, we discovered a ZIP code of only ~2 Hectares in size and it designates just a single, small private university and was inside another ZIP code. As a result of peculiarities like this and others, the number of total ZIP codes stated for Arkansas can vary. However, we were careful to geocode a contiguous single ZIP code layer that was consistent with Mtb case recording. This resulted in a spatially referenced layer of 579 ZIP codes.

We employed surface-interpolation analysis. One of the great analytical benefits of geographically referenced datasets is that they can be interpolated into surfaces [1]. Surface modelling techniques capture spatial relationships of location, attribute data that can reveal important patterns of locational similarity and dissimilarity [1][2]. Such analyses, then, can be layered onto other spatial datasets that can then be assessed.

Kriging is a non-linear surface interpolation technique that conducts a preliminary assessment of spatial correlation and variation (semivariogram) in the spatial data to indicate a best-fit model that can then result in probability or predictive surfaces [2][3]. Semivariograms calculate the average degree of spatial dissimilarity of the attributes of interest [3]. We employed co-kriging, which utilizes correlated variables to better estimate the distribution of the dependent variable.

Our GIS spatial, exploratory regression approach searched for candidate explanatory variables for models with at least the minimum number of explanatory variables (1) and not more than the maximum number (5) that best explain the variation found in the dependent variable.

For variables that would not be directly related to or covary with other variables (e.g., median income vs total population), we normalized raw data by using the highest value in the array as the divisor for all values, producing a dataset with values from 0 to 1. For variables that were subsets of parent values (i.e., race populations vs total population) we calculated their proportions. A third method was log transformation. The total number of TB cases recorded per ZIP code over the study period ranged from 0 to 114; however, over 92% of the ZIP codes recorded 20 or fewer TB cases (43% 1 or less cases) with the second highest observed number of cases being 27. Due to non-uniform distributions with values concentrated at low values, we log transformed the total number of observed cases per ZIP code.

**References**

1. Jones, C. 1997. *Geographical information systems and computer cartography*. Addison-Wesley-Longman; Essex, England. 329pgs.
2. Butler. J. R. (2003). The Spatial Impact of an Urban Area on breeding birds.*J. of the Tennessee Academy of Science.* 78(4), 124-141.
3. Goovaerts, P. (1997). *Geostatistics for natural resources evaluation*. Oxford University Press, New York. New York. 496pgs.
